# A clinical report of the massive CAG repeat expansion in spinocerebellar ataxia type 2: Severe onset in a Mexican child and review previous cases

**DOI:** 10.1590/1678-4685-GMB-2019-0325

**Published:** 2020-08-21

**Authors:** José Sánchez-Corona, Sergio Alberto Ramirez-Garcia, Gema Castañeda-Cisneros, Susan Andrea Gutiérrez-Rubio, Víctor Volpini, Diana M. Sánchez-Garcia, José Elías García-Ortiz, Diana García-Cruz

**Affiliations:** ^1^ Instituto Mexicano del Seguro Social - IMSS, Centro de Investigaciones Biomédicas de Occidente - CMNO, División de Medicina Molecular, Jalisco, Mexico.; ^2^ Universidad de la Sierra Sur, Instituto de Nutrición y de Investigaciones sobre la Salud Pública, Oaxaca, Mexico.; ^3^ Instituto Mexicano del Seguro Social – IMSS, UMAE Hospital de Especialidades, Centro Médico de Occidente, Guadalajara, Jalisco, Mexico.; ^4^ Universidad de Guadalajara, Centro Universitario de Ciencias de la Salud - CUCS, Departamento de Fisiología, Guadalajara, Jalisco, Mexico.; ^5^ Institut d'Investigació Biomédica de Bellvitge - IDIBELL, Centro de Diagnóstico Genético Molecular, Barcelona, Spain.; ^6^ ITESO, Universidad Jesuita de Guadalajara, Guadalajara, Jalisco, Mexico.; ^7^ Instituto Mexicano del Seguro Social - IMSS, Centro de Investigación Biomédica de Occidente - CIBO, División de Genética, Guadalajara, Jalisco, Mexico.; ^8^ Universidad de Guadalajara, Centro Universitario de Ciencias de la Salud - CUCS, Instituto de Genética Humana “Enrique Corona Rivera”, Jalisco, Mexico.

**Keywords:** Ataxin-2 gene, anticipation phenomenon, autosomal dominant, spinocerebellar ataxia type 2, triplet expansion repeat

## Abstract

The spinocerebellar ataxia type 2 is a neurodegenerative disease with autosomal dominant inheritance; clinically characterized by progressive cerebellar ataxia, slow ocular saccades, nystagmus, ophthalmoplegia, dysarthria, dysphagia, cognitive deterioration, mild dementia, peripheral neuropathy. Infantile onset is a rare presentation that only has been reported in four instances in the literature. In the present work a boy aged 5 years 7 months was studied due to horizontal gaze-evoked nystagmus, without saccades, ataxic gait, dysarthria, dysphagia, dysmetria, generalized spasticity mainly pelvic, bilateral Babinsky. The mother aged 27 years-old presented progressive cerebellar ataxia, dysarthria, dysmetria, dysdiadochokinesis, limb ataxia and olivopontocerebellar atrophy. The molecular analysis was made by identifying the expansion repeats in tandem by long PCR to analyze the repeats in the ATXN2 gene. We found an extreme CAG expansion repeats of ~884 repeats in the child. We describe a Mexican child affected by SCA2 with an infantile onset, associated with a high number of CAG repeats previously no reported and anticipation phenomenon.

Spinocerebellar ataxia type 2 (SCA2) (MIM ID #183090) is an autosomal dominant disorder with progressive cerebellar ataxia, slow ocular and dysmetric vertical saccades, gaze-evoked nystagmus, supranuclear ophthalmoplegia, dysarthria, dysphagia, rigidity, spasticity, dysmetria and dysdiadochokinesis, bradykinesia, myoclonus, cognitive deterioration, mild dementia, dopamine-responsive parkinsonism, peripheral neuropathy and olivopontocerebellar atrophy (Choudhry *et al.*, 2001). SCA2 has a global prevalence of 1:35,000 individuals, is caused by (CAG)n abnormal long expansions in the ataxin-2 gene (*ATXN2*, 601517) (gene map locus 12q24.12), encoding a polyglutamine tract (polyQ) in the mutant protein which represent the large majority.

SCA2 is the most prevalent of the polyQ ataxias in Cuba, India, Mexico, and Southern Italy, and the second most prevalent worldwide, accounting for 15% of all cases (Antenora *et al.*, 2017). An infantile onset is an uncommon occurrence presentation with only nine reports in the literature and is associated with extremely large CAG expansions (range 100–200 repeats). On the other hand, there are severe infantile cases with expansions between 50-70 repeats, sometimes is related with interruption CTG or CAA as occurs in adults with SCA2 and amyotrophic lateral sclerosis, which also leads to instability of *ATXN2* mRNA (Choudhry *et al.*, 2001; Antenora *et al.*, 2017), and meiotic instability a general feature of SCA2 without a familial history (Babovic *et al.*, 1998; Mao *et al.*, 2002; Moretti *et al.*, 2004; Dirik *et al.*, 2007; Abdel and Zaki, 2008; Paciorkowski *et al.*, 2011; Di Fabio *et al.*, 2012; Vinther-Jensen *et al.*, 2013; Singh *et al.*, 2014). However, cases with infantile SCA2 are explain by these features; there are childhood onset cases reported with mosaicism in germ cells such as spermatozoa with larger expanded alleles more than in peripheral blood cells (Moretti *et al.*, 2004; Dirik *et al.*, 2007; Abdel and Zaki, 2008; Vinther-Jensen *et al.*, 2013).

The most frequent signs in infantile onset are developmental delay, visual impairment usually dependent on retinitis pigmentosa or optic atrophy, hypotonia, seizures with infantile spasms or myoclonic seizures, facial dysmorphism, dystonic features and early death (Tables 1 and 2) (Singh *et al.*, 2014; Antenora *et al.*, 2017). Brain MRI scans showed extreme cerebellar and brainstem atrophy, but also different degrees of supratentorial atrophy, ventricular enlargement, and white matter signal abnormalities probably attributable to dysmyelination and/or delayed myelination (Singh *et al.*, 2014; Antenora *et al.*, 2017). The aim of this report is to present the clinical findings and molecular studies in a Mexican child with familial SCA2 with extremely large CAG expansion repeats, and a literature review of clinical and molecular findings in early-onset cases with SCA2.

**Table 1:** Previous cases review of infantile-onset SCA2.

Reference	Main findings	Age of onset	Genotype *ATXN2* (CAG repeats)	Parental origin *ATXN2*(CAG repeats)	Age of death
Babovic *et al*, 1998	Child was born with neonatal hypotonia, at 2 wk progressive apnea, developmental delay, and dysphagia, Retinitis pigmentosa was noted at 10 mo. Next 7 mo, the child remained hypotonic with increase feeding difficulties, dysphagia, and more frequent apneas. The child continued to show slow progression of her neurological symptoms and died from respiratory complications.	Newborn	23/~220	Father 22/43	2 yr
Mao *et al*, 2002	Male with severe hypotonia, 2 mo; poor head control, Poor visual alertness; no retinopathy; intermittent esotropia. At 7 mo: marked cerebellar atrophy, delayed myelination. EEG: hypsarrhythmia at 14 mo	11 mo	22/230	Father 22/40	22 mo
Mao *et al*, 2002	Male with hypotonia, developmental delay and dysphagia, Retinitis pigmentosa. MRI At 3 mo: normal. EEG: normal at 3 mo.	10 mo	22/400	Father 22/43	2 yr
Mao *et al*, 2002	Male with encephalopathy, chronic seizures, hypertonic extremities, chronic seizures, axial, hypotonia, severe developmental retardation, microcephaly, short stature, Visual impairment, In MRI Cerebellar atrophy, EEG: hypsarrhythmia	3 mo	22/350	Father 22/40	NR
Mao *et al*, 2002	Male with hypotonia, visual impairment, delayed motor development, nystagmus, dysconjugated, gaze, convergent, strabismus, pigmentary, retinopathy, MRI at 7 mo: mild diffuse parenchymal loss, delayed myelination. At 18 mo: moderate diffuse cerebellar atrophy, EEG repetitive discharges of sharp and slow wave activity; pronounced slowing of EEG	10 mo	22/500	Mother 22/45	NR
Moretti *et al*, 2004	A male child initially presented with abnormal eye movements at age 2 mo, developmental delay at 6 mo. At the 7 yr he developed ataxia and cognitive impairment, and subsequently manifested dysphagia and incontinence. At age 11 yr, he had bilateral external ophthalmoplegia, ataxic dysarthria, dysmetria and tremor in the upper extremities, and marked gait ataxia, and brain MRI demonstrated cerebellar, brainstem, and cerebral atrophy.	2 mo	22/62	Mother 22/22. Father non tested.	NR
Dirik *et al*, 2007	A female baby appeared completely normal at birth. At the age of 5 yr, began ataxia, dysarthria, head titubation, and cognitive deficits. At age 8 yr, she was not able to walk because of severe ataxia. She also had drooling, feeding problems, and bladder dysfunction. Severe truncal ataxia, tremor, dysarthria, dysmetria, hyporeflexia, and slow saccades.	5 yr	22/70	Father 22/40	NR
Abdel *et al.*, 2008	Proband male starting as early as 2 yr old with progressive extrapyramidal manifestations, slow eye movements and cognitive impairment. The patient lost all cognitive functions, had persistent dystonic, vasomotor instability, and dysphagia and died at the age of 7 yr. The early neurological symptoms included choreoathetotic, myoclonic jerk, gait difficulty, and emotional liability, ataxia, incoordination, dysarthria, mild dementia and slow eye saccades predominated. Peripheral neuropathy, polyphagia and obesity.	24 mo	22/ 69 to 75	Father 22/ 39	7 yr
Paciorkowski *et al*, 2011	This infant girl presented with apnea at 2 wk, occipital-frontal circumference was 3 SD at 10 mo of age, Retinitis pigmentosa, developed infantile spasms at 16 mo, with hypsarrhythmia on EEG. She died at two yr old from respiratory complications.	2 wk	22/220	Mother 22/43	2 yr
Paciorkowski *et al*, 2011	This infant girl presented hypotonia at 6 mo, optic nerve atrophy, cerebellar atrophy and delayed myelination and enlarged lateral ventricles, and obvious cerebellar and brainstem atrophy. At 14 mo she developed infantile spasms with hypsarrhythmia. She had relative microcephaly and optic nerve atrophy. Death at 22 mo due to aspiration pneumonia.	6 mo	22/200	Father 22/42	22 mo
Paciorkowski *et al*, 2011	This infant boy presented at 3 mo with focal seizures. At 10 mo he developed infantile spasms with hypsarrhythmia. Brain MRI at 10 mo showed prominent sulci frontally, enlargement of the lateral and third ventricles, and an atrophic-appearing cerebellum. At 12 mo he was microcephaly, diagnosed cortical visual impairment. He had impaired swallowing and autonomic instability. He died at 13 mo.	3 mo	22/ > 200	Mother 22/45	13 mo
Paciorkowski *et al*, 2011	This infant girl presented with loss of head control and poor visual fixation at 3 mo and at 5 mo she had myoclonic seizures. At 17 mo EEG showed high-amplitude bursts of slow waves with polyspikes reminiscent of hypsarrhythmia. Brain MRI at 6 mo showed diffuse parenchymal volume loss and delayed myelination. She was microcephaly and by 21 mo with minimal visual interaction and retinitis pigmentosa. She died at 32 mo.	3 mo	22/500	Father 22/40	32 mo
Paciorkowski *et al*, 2011	This infant girl had global developmental delay in infancy, and microcephaly. At 4 yr she was failing to thrive with poorly coordinated swallow, at the 10 mo later began to lose motor and cognitive milestones and had autonomic dysfunction, optic nerve atrophy and retinitis pigmentosa, brain MRI at 5 yr 10 mo showed diffuse T2 white matter signal abnormalities, with cavitations of the parietooccipital lobes and cortical and cerebellar volume loss.	48 mo	22/750	Father 22/40	NR
Paciorkowski *et al*, 2011	This infant girl presented at 2 mo with poor head control and lack of visual fixation, at 7 mo she had tonic seizures, EEG at 14 mo showed multifocal epileptiform discharges. At 12 mo with retinitis pigmentosa. Brain MRI at 12 months showed mild cerebellar atrophy. The patient died after a neurodegenerative course. Pathologic examination of the cerebellum showed profound loss of Purkinje and granular neurons with severe attenuation of the molecular layer.	2 mo	22/300	Father 22/43	NR
Di Fabio *et al*, 2012	A girl who presented with facial dysmorphism, dystonic features, developmental delay, and retinitis pigmentosa; and associated with developmental delay and retinitis pigmentosa in early childhood.	12 mo	22/92	Father 22/51	
Vinther-Jensen *et al.*, 2013	Male and his daughter aged 6 mo, she was referred to a local hospital with uncoordinated eye movement with parallel eye axes, lack of head control and hypotonia in the upper extremities and the trunk, generalized myoclonic jerks and athetoid movements. EEG showed bilateral spike foci in the frontal and parietal regions. At the age of 9 mo, brain MRI was normal except for a relatively large cerebellum-medullary cistern. At the 13 mo, she had motor improvement, dyskinesia, delayed visual development, pallor of the optic nerves and hyperpigmentation of dystrophic retina. At the 17 mo, generalized edema and proteinuria, and minimal change glomerulonephritis. She died of sepsis and multi-organ failure 2 mo later. His brother had gait disturbances, and his daughter had died from multi-organ failure at age 19 mo.	6 mo	Daughter 124 CAG repeats, Brother single-cell sperm in 92, in genomic DNA 45 CAG repeats	Father 22/45	19 mo
Singh *et al*, 2014	A 10-month male child was referred to regression of milestones since 6 mo of age. Here was no obvious dysmorphology, decreased muscle tone, inability to roll in his bed, and decreased tendon reflexes in all limbs and retinitis pigmentosa. MRI (at age of 10 mo) showed a markedly small cerebellum and vermis with associated atrophy involving the brainstem and both cerebral hemispheres. There was massive enlargement of the ventricles and enlarged infratentorial subarachnoid spaces. Additionally, diffuse T2 hyperintensity was observed within the periventricular white matter. Arachnoid cysts within the posterior fossa and right anterior temporal region were incidental findings.	6 mo	22/*320	Father 22/47	NR
Avelino *et al.*, 2014	A 1-year-old Brazilian girl presented the phenotype of very early-onset SCA2 (neonatal form) encephalopathy, with hypotonia, choreic movements, dystonia with dystonic jerks, seizures, and retinitis, motor developmental delay. During the neonatal period (first mo), she presented hypotonia, dysphagia, and frequent gastroesophageal reflux. Tonic seizures, hypotonic with increased feeding difficulties, and choreic movements and dystonia with dystonic jerks. Decreased tonus and global decreased tendon reflexes. Visual fixation was poor and erratic eye, ophthalmoplegia were observed. Fundoscopy disclosed abnormal retina, with white dots, suggesting retinitis punctata albescens. Brain MRI showed marked cerebellar and brainstem atrophy and mild delayed myelination.	Newborn	22/104	Father Non determinated	NR

NR – not recorded; mo- months, yr- years, wk-weeks.

**Table 2:** Clinical Epidemiology of the findings in infantile-onset SCA2.

Clinical findings	Case	Mother	Total reported cases
**Neurological**			
Hypotonia	-	-	8/19
Tremor	-	-	19/19
Ataxia	+	+	19/19
Dysmetria	-	+	19/19
Dysdiadochokinesia	-	+	19/20
Dysarthria	+	+	19/19
Neurological and cognitive deterioration	+	-	3/19
Saccades	-	-	2/19
Abnormalities in the electroencephalogram	-	+	11/19
**MRI or CT**			
Cerebellar and brainstem atrophy	-	-	12/19
Olivopontocerebellar atrophy	+	+	19/19
Atrophy involving both cerebral hemispheres	-	-	2/19
Massive enlargement of the ventricles and enlarged infratentorial subarachnoid spaces	-	-	3/19
Arachnoid cysts within the posterior fossa and right anteriortemporal region; relatively large cerebellum and medullary cistern	-	-	2/19
Diffuse T2 white matter signal abnormalities	-	-	3/19
Cavitations of the parieto occipital lobes and cortical and cerebellar volume loss	-	-	1/19
Prominent sulci frontally and delayed myelination	-	-	1/19
**Ophthalmological**			
Visual alertness	-	-	7/19
Intermittent esotropia; Visual impairment	-	-	2/19
Nystagmus	+	-	3/19
Dysconjugated gaze, convergent strabismus, ophthalmoplegia	-	-	2/19
Atrophy of the optic nerve	-	-	2/19
Incoordinated eye movement with parallel eye axes	-	-	1/19
Retinitis pigmentosa	-	-	11/19
Retinitis punctata albescens	-	-	1/19
**Miscellaneous**			
Microcephaly	-	-	4/19
Facial dysmorphism	-	-	1/19
Peripheral nerve affection	+	-	1/19
Polyphagia	-	-	1/19
Macrosomia	+	-	1/19
Pyramidal failure pathway	-	-	1/19
Generalized edema	-	-	1/19
Proteinuria	-	-	1/19
Minimal change glomerulonephritis	-	-	1/19
Problems or difficulty feeding	+	-	2/19
Urinary incontinence	+	-	2/19
Gastroesophageal reflux	-	-	1/19
Dysphagia	+	-	5/19

Notes. Abnormalities in the electroencephalogram are considered hypsarrhythmia, repetive discharges of sharp and slow wave activit; pronounced slowing, infantile spasms with hypsarrhythmia, highamplitude bursts of slow waves with polyspikes reminiscent of hypsarrhythmia, multifocal epileptiform discharges, bilateral spike foci in the frontal and parietal regions and seizures. MRI = magnetic resonance imaging; CT = computed tomography.

The clinical findings in the index case, a boy aged 5 yr 7 mo (Figure 1A), was the product of the first pregnancy complicated by hyperemesis during the first trimester, delivery was by cesarean section due to macrosomia. Since birth the patient presented feeding difficulties due to dysphagia that persists at this time; at 3 months of age nystagmus was present. Psychomotor development, at 12 months he began to walk with evident ataxic gait. When he started speaking at 2-3 years of age, he presented dysarthria, at this moment he has progression of all the symptomatology, poor coordination, walking difficulty, incontinence (he never had sphincter control) and he continues with dysphagia. At physical examination he presented horizontal gaze-evoked nystagmus, without saccades, ataxic gait, dysarthria, dysphagia, dysmetria, generalized spasticity mainly pelvic, bilateral Babinsky. CT brain scan revealed mild pontine atrophy, moderate cerebellar atrophy (Table 2) (Figure 1B,C).

**Figure 1:**
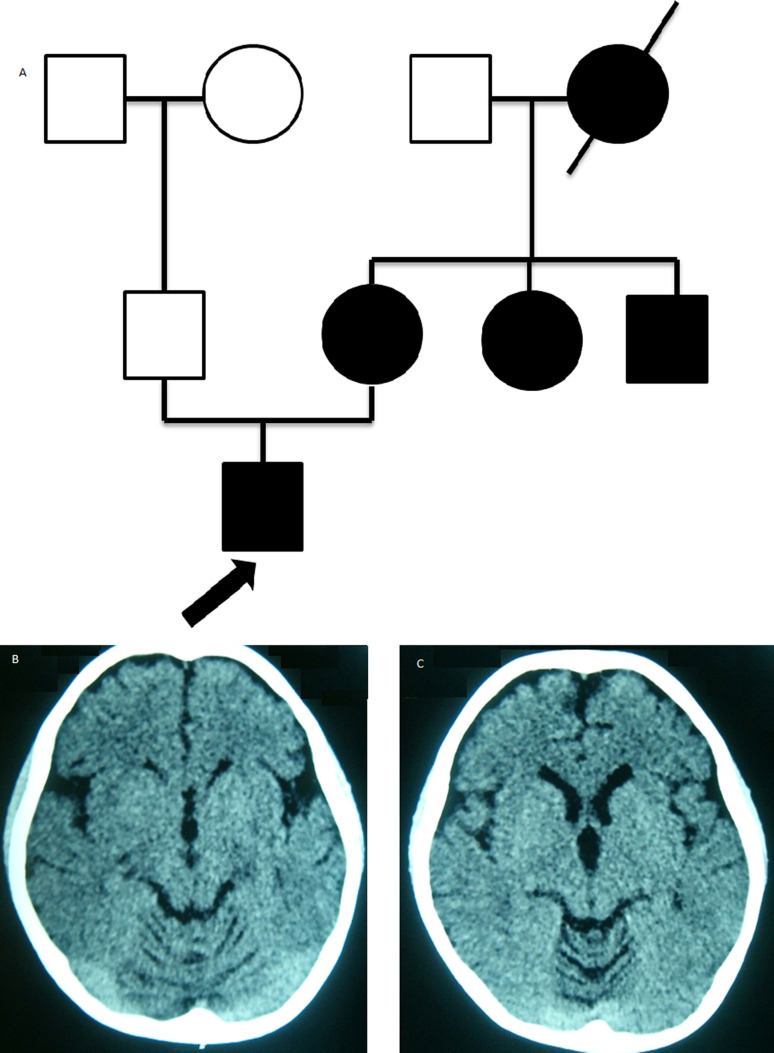
A. Pedigree, arrow: the *propositus.* B-C*., Propositus'* CT brain scan showing mild pontine atrophy and moderate cerebellar atrophy.

Family data: Mother aged 27 years similarly affected, clinical symptomatology began at 7 years of age with walking and speech difficulties. At 10 years of age she started with seizures, controlled for 5 years. Currently presents ataxia, dysarthria, dysmetria, dysdiadochokinesia, ataxic gait with olivopontocerebellar atrophy. Father aged 30-year-old neurologically healthy. Two maternal aunts of 30 and 23 years and the maternal grandmother (age of onset 40 yr), were similarly affected. Mother with molecular analysis positive to SCA2 with a genotype 22/49 CAG expansion repeats and father with normal range or expansion repeats.

Genomic DNA was extracted from peripheral blood leukocytes using the Gene Catcher Kit (Invitrogen) of the index case. The analysis of the ataxin-2 gene was performed by long PCR using a pair of oligonucleotides flanking the repeated CAGs. The sequence of the oligonucleotides was as follows: FWP1 5′-GGGCCCCTCACCATGTCG-3′; and RWP 5′-CGGGCTTGCGGACATTGG-3′ (Sigma Aldrich). PCR was carried out in a total volume of 10 μl containing 100 ng of human DNA, 0.4 μM of each oligonucleotide, 200 μM of each dNTP; 0.6 μl of the 10x reaction buffer (Roche Diagnostics GmbH), 2 mM MgCl2 (Roche Diagnostics), 0.5 U of the enzyme *Taq* DNA polymerase (Roche Diagnostics GmbH) and 10% DMSO (Sigma-Aldrich) (Magaña *et al.*, 2008; Flores-Alvarado *et al.*, 2016). The amplification program consisted of 28 cycles, including denaturation at 96 °C for 60 s, hybridization at 59 °C for 30 s and polymerization at 72 °C for 60 s (Magaña *et al.*, 2008; Flores-Alvarado *et al.*, 2016).

The PCR product was mixed with deionized formamide and denatured in water bath for seven minutes. Then it was placed on ice frappe for five minutes, and subjected by electrophoresis on 10% polyacrylamide gels (PAGE, 29:1). From the PCR products, were made a dilution 1:5 to apply 5 mL in the respective lane. PAGE gel was run at 150 volts, during 6.25 hours (Magaña *et al.*, 2008); and subsequently stained with a solution containing; 0.1 g silver nitrate, 0.5 mL of acetic acid and 10 mL of ethanol graduated at 100 mL (12). The development solution used in the PAGE gels contained 3% sodium hydroxide with 270 μL of 37% formaldehyde graduated to 100 ml (Flores-Alvarado *et al.*, 2016).

The amplified fragment of 130 bp corresponds to a normal allele of 22 CAG repeats. The normal range is between 13-31 CAG repeats and in affected cases greater than 32 repeats (Guzmán-López *et al.*, 2018). In the PCR-PAGE denaturing assay (Figure 2) the first lane is a molecular weight marker of 100 bp, the second lane is the white control PCR which appears white, without amplified product, in lanes 4 and 6 is the sample 124 with a DNA dilution of 1:5, there is a band of 130 bp and another band at 2 kb that was calculated as 2,652 bp which generates the genotype 22/~884 repeats; in lanes 3 and 7 is the sample 125 with a DNA dilution of 1:5, a band of 130 bp is observed and in lane 5 there is a negative control sample for the trinucleotide expansion repeat. Thus, with these considerations, the index case presented a normal allele of 22 CAG repeats, and an allelic variant of ~884 CAG expansion repeats (2,652 bp), being carrier of the genotype 22/~884. This result was corroborated by the RED (Repeat Expansion Detection) method in the IDIBELL Center of Molecular Diagnosis; showing a higher penetrance allele of 2600 bp.

**Figure 2:**
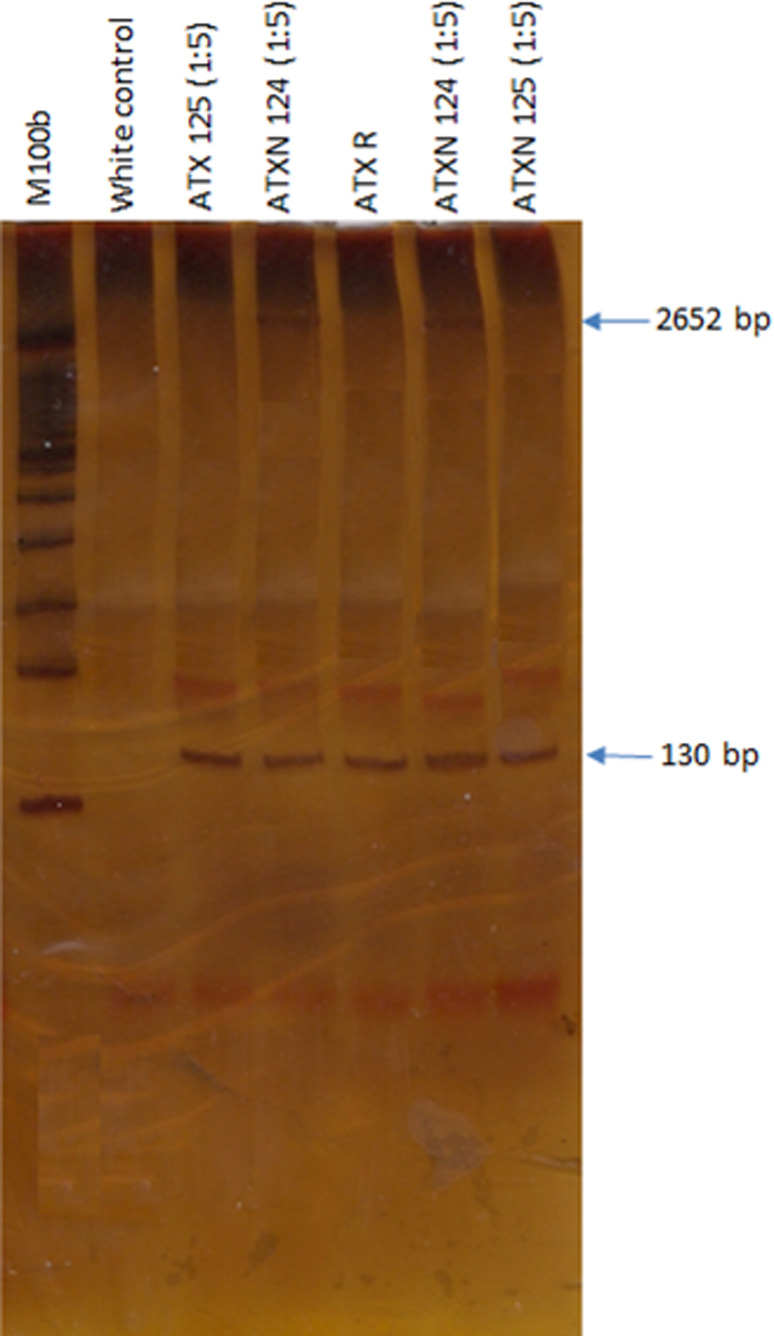
PAGE gel showing in the 4^th^ and 6^th^ lanes the extreme CAG repeats expansion ~884.

The present case is the first report of an index case with autosomal dominant SCA2 of neonatal presentation due to massive expansion of more than 884 CAG repeats in exon 1 of *ATXN2*. Massive expansions in the SCA2 are considered above one hundred repeats, longer are associated with an early onset age, and also are related with severe clinical findings (Antenora *et al.*, 2017). Certainly, the present case started with symptoms since birth and severe neurological deterioration and a genotype with a longer allele, compared with other neonatal cases (Babovic *et al.*, 1998; Dirik *et al.*, 2007).

So, the range in 19 reported cases with SCA2 with age of onset in childhood was 0-48 months, which were carriers of the heterozygous genotype (22/X), in which X corresponds to an allele with abnormal expansion repeats with an average range of 62-841 repeats (Table 1) (Babovic *et al.*, 1998; Mao *et al.*, 2002; Moretti *et al.*, 2004; Dirik *et al.*, 2007; Abdel and Zaki, 2008; Paciorkowski *et al.*, 2011; Di Fabio *et al.*, 2012; Vinther-Jensen *et al.*, 2013; Avelino *et al.*, 2014; Singh *et al.*, 2014).

Abnormal expansions in many trinucleotide expansion diseases are related with the anticipation phenomenon, resulting into longer mutant alleles of maternal origin. However, in infantile SCA2 with massive expansion, most of the reported cases with paternal origin contribute with a greater effect to the anticipation phenomenon (Table 1). On the other hand, there are three reported cases and the present one, with a maternal origin associated with a massive expansion (Mao *et al.*, 2002; Paciorkowski *et al.*, 2011; Trang *et al.*, 2015).

There are two clinical prognostic severity markers in SCA2, which are saccades and dysphagia (Wadia *et al.*, 1998). Dysphagia was reported in five cases with massive repeats, greater than 200 repeats (Babovic *et al.*, 1998; Mao *et al.*, 2002; Abdel and Zaki, 2008; Avelino *et al.*, 2014). The present case presented dysphagia since birth.

Abnormalities in the electroencephalogram were frequent in infantile SCA2 cases reports, but the present case did not present abnormal patterns in the electroencephalogram or epilepsy with tonic seizures or myoclonic seizures (Mao *et al.*, 2002; Paciorkowski *et al.*, 2011; Vinther-Jensen *et al.*, 2013; Singh *et al.*, 2014). While cerebellar and brainstem atrophy were related with massive CAG expansions (> 200) in the *ATXN2* gene (Babovic *et al.*, 1998; Mao *et al.*, 2002; Moretti *et al.*, 2004; Dirik *et al.*, 2007; Paciorkowski *et al.*, 2011; Magaña *et al.*, 2008; Vinther-Jensen *et al.*, 2013; Singh *et al.*, 2014; Avelino *et al.*, 2014). A case whose brain at 5 years 10 months showed diffuse T2 white matter signal abnormalities, with cavitations of the parieto-occipital lobes and cortical and cerebellar volume loss, was found carrier of a genotype 22/~750 (Paciorkowski *et al.*, 2011). In the present case the CT brain scan revealed mild pontine atrophy, moderate cerebellar atrophy, with longer repeats 22/~841. This indicates that the severity of the damage to the brain structures not only depend of the *ATXN2* VNTR expansion, but may influence other genes in which *ATXN2* forms a network, that corresponds to a new frontier of research in SCA2.

The present case presented gaze-evoked nystagmus without retinitis pigmentosa common in childhood SCA2 due to long CAG repeats in *ATXN2* gene (see Table 2), greater than one hundred repeats (Babovic *et al.*, 1998; Mao *et al.*, 2002; Paciorkowski *et al.*, 2011; Di Fabio *et al.*, 2012; Vinther-Jensen *et al.*, 2013; Avelino *et al.*, 2014; Singh *et al.*, 2014). In the literature has been reported massive expansion repeats (more than 750 repeats) in SCA2 with autonomic dysfunction, retinitis pigmentosa, and infantile spasms; the difference in the present case without retinitis pigmentosa, not only be explained by the age of onset, but also by the same molecular and allelic heterogeneity of retinitis, which is polygenic with different inheritance patterns (Fahim *et al.*, 2018).

In patients with infantile SCA2, some craniofacial alterations were found, such as microcephaly in four cases and one case showed facial dysmorphism (Paciorkowski *et al.*, 2011). These findings were not relevant in the present case, but it would be worthwhile to analyze them clinically in detail, because there is a possibility that they are not syndromic but may be related to an *ATXN2* effect on cranial morphogenesis.

Peripheral nerve affection, polyphagia and obesity were striking manifestations in the middle stage of the disease, as well as generalized edema and proteinuria, and minimal change glomerulonephritis that were found in an isolated patient. This might support a previously suggested relationship between the ataxin-2 gene and body weight and insulin resistance, which is modulated by ataxin-2 by the pathway of insulin by GRB2 and SRC (Flores-Alvarado *et al.*, 2016; Guzmán-López *et al.*, 2018; Ramirez-Garcia *et al.*, 2019). The two carriers of these phenotypic findings had an expanded alleles range between 90 and less than 130 CAG repeats, which corroborates the participation of ataxin-2 gene in the development of metabolic syndrome traits (Flores-Alvarado *et al.*, 2016; Ramirez-Garcia *et al.*, 2019). However, our tester has the highest repeating range in the literature, and has not developed any of these findings, which suggests that there are other genes that can modify the SCA2 phenotype, especially in the case of children (Ramírez-Garcia *et al.*, 2011; Trang *et al.*, 2015).

In conclusion, the present report describes a child with infantile SCA2 with a massive expansion of CAG trinucleotides of the *ATXN2* gene reported up to date in the literature, which resulted in a very early onset of clinical findings, since birth, and whose phenotype mainly corresponds to the severe affection of the central and peripheral nervous system. Also, this is the first childhood case in a Mexican and Latin-American population. The review of the literature of 18 cases of infantile SCA2 and the comparison with the present case shows a great clinical and molecular heterogeneity, which does not always correlate with the greater number of CAG repeats of the *ATXN2* gene. The most frequent clinical signs were hypsarrhythmia, hypotonia, pigmentosa retinitis, cerebellar atrophy, as well as middle brain and lengthening of the ventricular system. The range of CAG repeats ranged between 69-884 repeats; however, longer repeats have more severe phenotypes.
